# 5-(4-Cyano-5-dicyano­methyl­ene-2,2-dimethyl-2,5-dihydro-3-fur­yl)-3-(1-methyl-1,4-dihydro­pyridin-4-yl­idene)pent-4-enyl 3,5-bis­(benz­yloxy)benzoate acetonitrile 0.25-solvate: a synchrotron radiation study

**DOI:** 10.1107/S1600536809050430

**Published:** 2009-11-28

**Authors:** Graeme J. Gainsford, M. Delower H. Bhuiyan, Andrew J. Kay

**Affiliations:** a Industrial Research Limited, PO Box 31-310, Lower Hutt, New Zealand

## Abstract

The title compound, C_42_H_36_N_4_O_5_·0.25CH_3_CN, crystallizes with a partial twofold disordered (1/4) acetonitrile solvent of crystallization. The linking atoms to the 3,5-bis­(benz­yloxy)benzoic acid are disordered between two conformations in the ratio 0.780 (6):0.220 (6). In the crystal, the mol­ecules pack using mainly C—H⋯N(cyano) inter­actions coupled with weak C—H⋯O(ether) inter­actions and C—H⋯π inter­actions. A brief comparison is made between a conventional and this synchrotron data collection.

## Related literature

For general background, see Kay *et al.* (2004 ▶); Marder *et al.* (1993 ▶). For related structures, see: Kay *et al.* (2008 ▶), Gainsford *et al.* (2007 ▶, 2008 ▶); Kim *et al.* (2007 ▶) For synthetic data, see: Clarke *et al.* (2009 ▶). For details of the PX1 beamline, see: McPhillips *et al.* (2002 ▶).
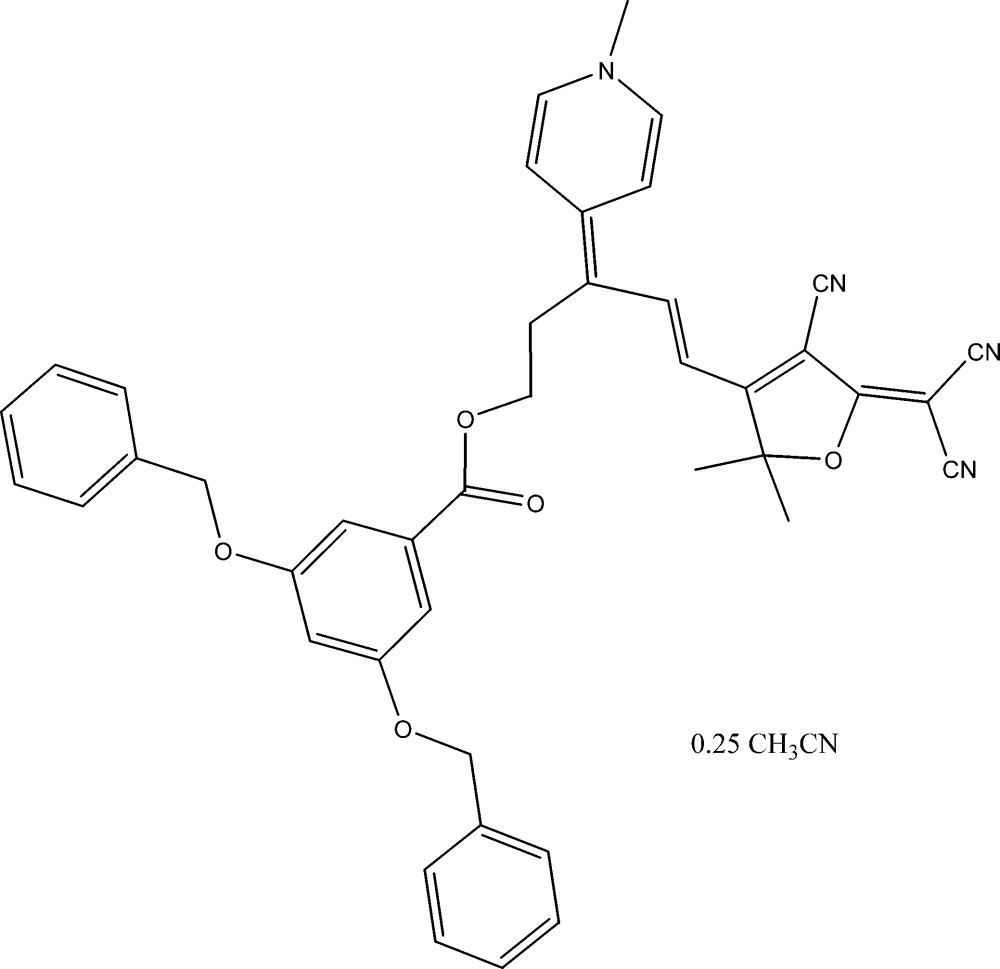


## Experimental

### 

#### Crystal data

C_42_H_36_N_4_O_5_·0.25C_2_H_3_N
*M* *_r_* = 687.01
Monoclinic, 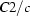

*a* = 29.374 (6) Å
*b* = 15.825 (3) Å
*c* = 16.317 (3) Å
β = 108.61 (3)°
*V* = 7188 (3) Å^3^
*Z* = 8
Synchrotron radiation
λ = 0.77300 Å
μ = 0.08 mm^−1^
*T* = 100 K
0.26 × 0.08 × 0.04 mm

#### Data collection

ADSC Quantum 210r CCD diffractometer
38733 measured reflections
5381 independent reflections
4076 reflections with *I* > 2σ(*I*)
*R*_int_ = 0.098
θ_max_ = 26.0°

#### Refinement

*R*[*F* ^2^ > 2σ(*F* ^2^)] = 0.060
*wR*(*F* ^2^) = 0.167
*S* = 1.04
5381 reflections
482 parameters
H-atom parameters constrained
Δρ_max_ = 0.91 e Å^−3^
Δρ_min_ = −0.50 e Å^−3^

### 

Data collection: *ADSC Quantum 210r software* (ADSC, 2009 ▶); cell refinement: *XDS* (Kabsch, 1993 ▶); data reduction: *XDS*, locally modified software and *XPREP* (Bruker, 2001 ▶); program(s) used to solve structure: *SHELXS97* (Sheldrick, 2008 ▶); program(s) used to refine structure: *SHELXL97* (Sheldrick, 2008 ▶); molecular graphics: *ORTEP-3* (Farrugia, 1997 ▶) and Mercury (Macrae *et al.*, 2006 ▶); software used to prepare material for publication: *SHELXL97* and *PLATON* (Spek, 2009 ▶).

## Supplementary Material

Crystal structure: contains datablocks global, I. DOI: 10.1107/S1600536809050430/sj2693sup1.cif

Structure factors: contains datablocks I. DOI: 10.1107/S1600536809050430/sj2693Isup2.hkl

Additional supplementary materials:  crystallographic information; 3D view; checkCIF report

## Figures and Tables

**Table 1:** Hydrogen-bond geometry (Å, °)

*D*—H⋯*A*	*D*—H	H⋯*A*	*D*⋯*A*	*D*—H⋯*A*
C9—H9*A*⋯N1^i^	0.98	2.59	3.529 (4)	161
C9—H9*B*⋯N3^ii^	0.98	2.59	3.497 (5)	153
C16—H16⋯N1^iii^	0.95	2.51	3.406 (4)	156
C17—H17⋯N2^iv^	0.95	2.51	3.380 (4)	152
C19—H19*C*⋯N2^iv^	0.98	2.50	3.340 (4)	143
C26—H26⋯O5^v^	0.95	2.51	3.398 (4)	155
C8—H8*B*⋯*Cg*1^vi^	0.98	2.54	3.515 (3)	171

Symmetry codes: (i) 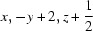; (ii) 
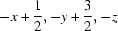
; (iii) 
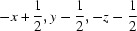
; (iv) 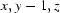; (v) 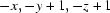; (vi) 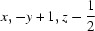. *Cg*1 is the centroid of the C30–C35 ring.

**Table 2:** Inter­planar angles and SIGP values for the planar entities (Å, °)

Plane	*P*1	*P*2	*P*3	*P*4	*P*5	SIGP^*a*^
*P*1		14.59 (10)	18.77 (7)	31.92 (12)	64.58 (13)	0.025 (3)
*P*2	14.59 (10)		4.90 (9)	18.33 (13)	75.25 (15)	0.033 (3)
*P*3	18.77 (7)	4.90 (9)		13.48 (11)	80.11 (12)	0.026 (3)
*P*4^*b*^	31.92 (12)	18.33 (13)	13.48 (11)		86.94 (16)	0.004 (3)

Notes: *P*1 = C1–C12,N1–N3,O1; *P*2 = C12–C19,N4; *P*3 = C22–C30,O3–O4; *P*4 = C29–C35; *P*5 = C37–C42. (*a*) 
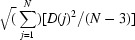
 (Spek, 2009 ▶); (*b*) SIGP for plane C37–C42 is 0.014 (3) Å.

**Fig. 1.**
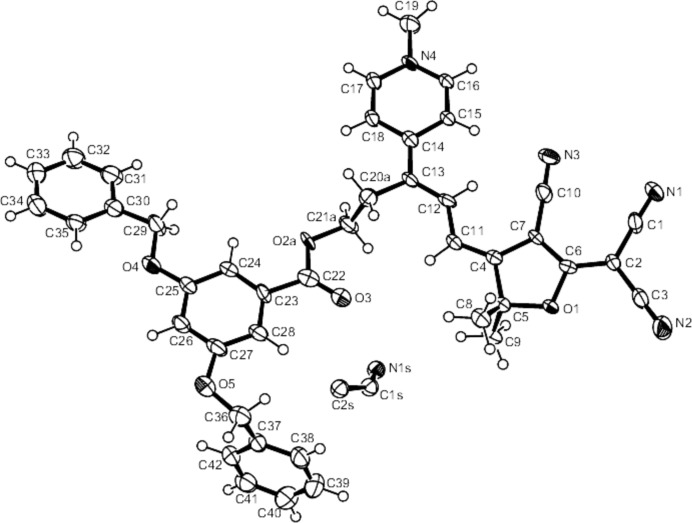
Molecular structure of the asymmetric unit (Farrugia, 1997); displacement ellipsoids are shown at the 50% probability level. Only the major conformation for atoms C20, C21 & O2 are shown; hydrogen atoms on the acetonitrile are omitted.

**Fig. 2.**
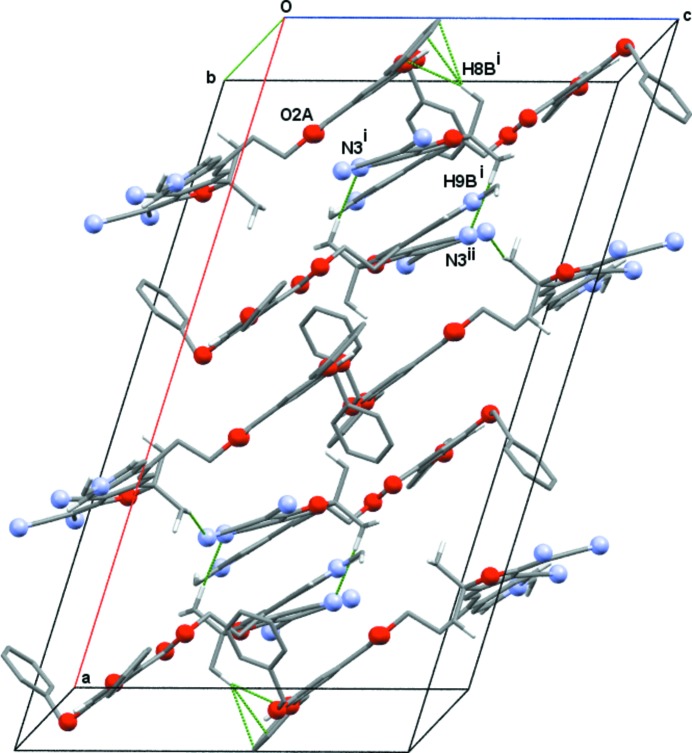
Packing diagram (Mercury, Macrae et al.,(2006)) of the unit cell Only H atoms involved in significant interactions are shown. Contact atoms are shown as balls; a limited set of labels are given (see Table 2). Symmetry codes: (i) x, 1 - y, 1/2 + z (ii) 1/2 - x, y - 1/2, 1/2 - z

## supplementary crystallographic information

### Comment

The title compound (I) was synthesized as part of a continuing research programme involving the development of organic nonlinear optical (NLO) chromophores. Due to the highly polar nature of these compounds they have a strong tendency to aggregate, a phenomenon that often leads to a reduction in the macroscopic nonlinearity. We have previously reported the crystallographic parameters for two chromophores containing a quinolinylidene donor coupled to a cyanodicyanomethylidenedihydrofuran (CDFP) electron acceptor group (Gainsford *et al.*, 2008). In both instances an examination of the unit cell packing showed plane to plane stacking of the chromophores *via* the interaction of the donor end of one molecule with the acceptor end of another. This is clear evidence for potentially deleterious H-aggregation occurring between the compounds. However, it has been reported that the introduction of bulky substituents near the centre of NLO chromophores leads to a significant reduction in the observed aggregation (Kim *et al.*, 2007). Consequently we were interested to make modifications to the core structure of our compounds to see whether the introduction of a bulky 3,5-bis(benzyloxy) benzoate group would lead to any change in the unit cell packing. We report here on the structural properties of a chromophore containing a pyridinylidene donor, CDFP acceptor and bulky side group. While a direct comparison between the compounds studied earlier (Gainsford *et al.*, 2008) and one also containing a quinolinylidene donor would be preferable, this wasn't feasible from synthetic viewpoint. An initial report based on the earlier conventional data collection has been given (Kay *et al.*, 2008).

The asymmetric unit of crystals of (I) contains one independent copy of the molecule and a partial (1/4) acetonitrile molecule disordered around a twofold symmetry site (Figure 1). The linking atoms to the 3,5-bis-benzyloxy-benzoic acid (C20,C21,O2) are disordered over two conformations with refined occupancies of 0.780:0.220 (6). The CDFP ring (atoms C4/C5/O1/C6/C7) and the cyano groups appended to C2 are coplanar (Table 2). The geometric parameters are consistent with the localized electron configuration shown in the scheme with ony subtle differences to the closely related quinoline molecules (NAJKUT & NOJLAA, Gainsford *et al.*, 2008); the similarity is reflected in the bond length alternation (Marder *et al.*, 1993) BLA values of -0.042 (here) and -0.015 & -0.042 respectively. The interplanar angles (Table 2) show the overall moleculear non-planarity of the adjacent planar entities.

The molecular packing is provided by mainly *C*–*H*···*N*(cyano) interactions but also a C–H···O interaction with one benzolyoxy oxygen and C—H ···π crosslinks (Figure 2). Two adjacent molecules are linked through the methyl hydrogen atoms (entries 1 & 2, Table 1) and extended into layers parallel to the (3,0,1) plane *via* the H16, H17 & H19C (entries 3,4 & 5) and the C–H···O5 (entry 6) interactions. These layers are crosslinked *via* methyl H8B interaction with phenyl ring C30—C35 (entry 7, Table 1; Figure 2).

An opportunity arose to recollect data on the Australian synchrotron, which is the data presented here. The conventional laboratory results are quite similar but that data did not support refinement of the partial acetonitrile solvent molecule. There were about 2.5 times more observed data in the synchrotron data set (collected in 5% of the conventional time) giving smaller su (by ~70%) values, but overall both datasets gave similar agreement factors. Given the overall agreement data statistics (see exptl_refinement) and the different crystal used, further comparison seems unwarranted.

### Experimental

The title compound was synthesized by a published procedure (Clarke *et al.*, 2009). Black crystals suitable for X-ray structure determination were grown in acetonitrile by slow evaporation of the solvent at room temperature.

### Refinement

A total of 12 outlier reflections were omitted from the processed set, from which 16 intense reflections with poor internal agreement had been removed. An examination of the data did not establish definitively that these latter data, with F_o_ >> F_c_, represented data from a multiple crystal fragment or were subject to twinning.

The acetonitrile solvent is disordered with the N atom on a 2 fold site; the occupancy was determined with an average isotropic U & then fixed at 0.25 with a common *U*_iso_ which refined to 0.082 (2) Å^2^. Atoms C20, C21 & O2 were found to be in two conformations which refined to occupancies of 0.780:0.220 (6); the minor conformer atoms (C20A,C20B & O2B) were refined isotropically to a common final value of 0.019 (2) Å^2^. The final difference minimum & maxima (-0.5 & 0.90 e/Å^-3^) are close to atoms N2S & C1S of the disordered acetonitrile carbon indicating imperfect modelling of the disorder.

All H atoms bound to carbon were constrained to their expected geometries (C–H 0.98, 0.99, 1.00 Å). Methyl H atoms were refined with *U*_iso_ = 1.5*U*_eq_(C); all other H atoms were refined with *U*_iso_ = 1.2*U*_eq_(C,*N*).

### Crystal data

**Table:**

C_42_H_36_N_4_O_5_·0.25C_2_H_3_N	*Z* = 8
*M**_r_* = 687.01	*F*(000) = 2892
Monoclinic, *C*2/*c*	*D*_x_ = 1.270 Mg m^−^^3^
Hall symbol: -C 2yc	Synchrotron radiation, λ = 0.77300 Å
*a* = 29.374 (6) Å	µ = 0.08 mm^−^^1^
*b* = 15.825 (3) Å	*T* = 100 K
*c* = 16.317 (3) Å	Needle, black
β = 108.61 (3)°	0.26 × 0.08 × 0.04 mm
*V* = 7188 (3) Å^3^	

### Data collection

**Table:**

ADSC Quantum 210r CCD diffractometer	4076 reflections with *I* > 2σ(*I*)
Radiation source: fine-focus sealed tube	*R*_int_ = 0.098
graphite	θ_max_ = 26.0°, θ_min_ = 1.6°
ω scans	*h* = −33→33
38733 measured reflections	*k* = −17→17
5381 independent reflections	*l* = −18→18

### Refinement

**Table:**

Refinement on *F*^2^	Secondary atom site location: difference Fourier map
Least-squares matrix: full	Hydrogen site location: inferred from neighbouring sites
*R*[*F*^2^ > 2σ(*F*^2^)] = 0.060	H-atom parameters constrained
*wR*(*F*^2^) = 0.167	*w* = 1/[σ^2^(*F*_o_^2^) + (0.0862*P*)^2^ + 13.1795*P*] where *P* = (*F*_o_^2^ + 2*F*_c_^2^)/3
*S* = 1.04	(Δ/σ)_max_ = 0.001
5381 reflections	Δρ_max_ = 0.91 e Å^−^^3^
482 parameters	Δρ_min_ = −0.50 e Å^−^^3^
0 restraints	Extinction correction: *SHELXL97* (Sheldrick, 2008), Fc^*^=kFc[1+0.001xFc^2^λ^3^/sin(2θ)]^-1/4^
Primary atom site location: structure-invariant direct methods	Extinction coefficient: 0.0128 (8)

### Special details

**Table:**

Geometry. All e.s.d.'s (except the e.s.d. in the dihedral angle between two l.s. planes) are estimated using the full covariance matrix. The cell e.s.d.'s are taken into account individually in the estimation of e.s.d.'s in distances, angles and torsion angles; correlations between e.s.d.'s in cell parameters are only used when they are defined by crystal symmetry. An approximate (isotropic) treatment of cell e.s.d.'s is used for estimating e.s.d.'s involving l.s. planes.
Refinement. Refinement of *F*^2^ against ALL reflections. The weighted *R*-factor *wR* and goodness of fit *S* are based on *F*^2^, conventional *R*-factors *R* are based on *F*, with *F* set to zero for negative *F*^2^. The threshold expression of *F*^2^ > σ(*F*^2^) is used only for calculating *R*-factors(gt) *etc*. and is not relevant to the choice of reflections for refinement. *R*-factors based on *F*^2^ are statistically about twice as large as those based on *F*, and *R*- factors based on ALL data will be even larger.

### Fractional atomic coordinates and isotropic or equivalent isotropic displacement parameters (Å^2^)

**Table:**

	*x*	*y*	*z*	*U*_iso_*/*U*_eq_	Occ. (<1)
O1	0.17400 (7)	0.94070 (11)	0.02908 (12)	0.0290 (5)	
O3	0.11459 (9)	0.63005 (13)	0.23553 (15)	0.0475 (7)	
O4	0.03753 (7)	0.32537 (12)	0.37018 (13)	0.0330 (5)	
O5	0.00478 (8)	0.60170 (12)	0.42819 (14)	0.0361 (5)	
N1	0.22209 (10)	0.92896 (14)	−0.22501 (16)	0.0329 (6)	
N2	0.19059 (12)	1.12873 (16)	−0.0685 (2)	0.0538 (9)	
N3	0.19800 (10)	0.73140 (14)	−0.16212 (16)	0.0324 (6)	
N4	0.21359 (9)	0.36102 (13)	−0.10782 (15)	0.0278 (6)	
C1	0.21038 (11)	0.94562 (16)	−0.16563 (19)	0.0256 (7)	
C2	0.19611 (11)	0.96943 (16)	−0.09403 (18)	0.0267 (7)	
C3	0.19290 (12)	1.05714 (18)	−0.0795 (2)	0.0354 (8)	
C4	0.17049 (10)	0.79170 (16)	0.02949 (17)	0.0241 (6)	
C5	0.16325 (11)	0.86922 (16)	0.07822 (18)	0.0255 (7)	
C6	0.18610 (10)	0.91040 (16)	−0.03864 (18)	0.0257 (7)	
C7	0.18566 (10)	0.82216 (16)	−0.04015 (17)	0.0234 (6)	
C8	0.11152 (12)	0.88153 (18)	0.0772 (2)	0.0331 (7)	
H8A	0.1025	0.8352	0.1088	0.050*	
H8B	0.0900	0.8818	0.0172	0.050*	
H8C	0.1088	0.9355	0.1047	0.050*	
C9	0.19856 (12)	0.87367 (17)	0.16911 (18)	0.0335 (8)	
H9A	0.1968	0.9296	0.1937	0.050*	
H9B	0.2312	0.8639	0.1673	0.050*	
H9C	0.1905	0.8304	0.2051	0.050*	
C10	0.19329 (11)	0.77332 (16)	−0.10791 (18)	0.0243 (7)	
C11	0.16310 (11)	0.71102 (16)	0.05218 (18)	0.0292 (7)	
H11	0.1496	0.7033	0.0974	0.035*	
C12	0.17441 (11)	0.63825 (16)	0.01213 (18)	0.0265 (7)	
H12	0.1922	0.6482	−0.0263	0.032*	
C13	0.16333 (13)	0.55628 (17)	0.0211 (2)	0.0395 (9)	
C14	0.18049 (11)	0.48951 (17)	−0.02358 (19)	0.0305 (7)	
C15	0.20573 (12)	0.50609 (17)	−0.08233 (19)	0.0331 (8)	
H15	0.2114	0.5630	−0.0947	0.040*	
C16	0.22209 (11)	0.44259 (16)	−0.12181 (19)	0.0305 (7)	
H16	0.2398	0.4560	−0.1598	0.037*	
C17	0.18892 (11)	0.34164 (16)	−0.05347 (18)	0.0282 (7)	
H17	0.1827	0.2841	−0.0444	0.034*	
C18	0.17278 (11)	0.40315 (17)	−0.01131 (19)	0.0294 (7)	
H18	0.1559	0.3875	0.0273	0.035*	
C19	0.23173 (14)	0.29362 (18)	−0.1516 (2)	0.0420 (9)	
H19A	0.2652	0.2809	−0.1183	0.063*	
H19B	0.2300	0.3125	−0.2098	0.063*	
H19C	0.2121	0.2427	−0.1559	0.063*	
C22	0.10633 (14)	0.55612 (19)	0.2443 (2)	0.0465 (9)	
C23	0.07702 (11)	0.52344 (18)	0.29680 (18)	0.0309 (7)	
C24	0.07207 (11)	0.43685 (18)	0.30567 (18)	0.0300 (7)	
H24	0.0867	0.3979	0.2776	0.036*	
C25	0.04542 (10)	0.40880 (17)	0.35614 (18)	0.0262 (7)	
C26	0.02430 (11)	0.46596 (18)	0.39724 (18)	0.0288 (7)	
H26	0.0064	0.4461	0.4326	0.035*	
C27	0.02924 (10)	0.55180 (17)	0.38689 (18)	0.0275 (7)	
C28	0.05616 (10)	0.58211 (18)	0.33736 (17)	0.0278 (7)	
H28	0.0602	0.6411	0.3313	0.033*	
C29	0.05841 (11)	0.26294 (18)	0.32904 (19)	0.0311 (7)	
H29A	0.0938	0.2683	0.3499	0.037*	
H29B	0.0471	0.2718	0.2657	0.037*	
C30	0.04418 (11)	0.17648 (18)	0.34973 (18)	0.0289 (7)	
C31	0.06809 (12)	0.10686 (19)	0.33084 (19)	0.0346 (7)	
H31	0.0930	0.1150	0.3062	0.041*	
C32	0.05569 (12)	0.02574 (19)	0.3477 (2)	0.0404 (8)	
H32	0.0723	−0.0214	0.3350	0.048*	
C33	0.01922 (12)	0.01313 (19)	0.3829 (2)	0.0400 (8)	
H33	0.0108	−0.0425	0.3946	0.048*	
C34	−0.00487 (12)	0.08144 (18)	0.4010 (2)	0.0344 (7)	
H34	−0.0301	0.0728	0.4248	0.041*	
C35	0.00742 (11)	0.16279 (18)	0.38470 (19)	0.0313 (7)	
H35	−0.0094	0.2096	0.3975	0.038*	
C36	0.01009 (12)	0.69126 (18)	0.4277 (2)	0.0373 (8)	
H36A	−0.0175	0.7178	0.4401	0.045*	
H36B	0.0091	0.7092	0.3690	0.045*	
C37	0.05594 (11)	0.72330 (17)	0.4923 (2)	0.0310 (7)	
C38	0.07674 (13)	0.79806 (19)	0.4777 (2)	0.0419 (9)	
H38	0.0645	0.8257	0.4234	0.050*	
C39	0.11551 (14)	0.8330 (2)	0.5421 (3)	0.0510 (10)	
H39	0.1291	0.8848	0.5317	0.061*	
C40	0.13430 (13)	0.7930 (2)	0.6206 (2)	0.0452 (9)	
H40	0.1604	0.8175	0.6647	0.054*	
C41	0.11505 (11)	0.71712 (19)	0.6349 (2)	0.0365 (8)	
H41	0.1287	0.6881	0.6880	0.044*	
C42	0.07586 (11)	0.68318 (17)	0.5719 (2)	0.0306 (7)	
H42	0.0623	0.6317	0.5831	0.037*	
O2A	0.12743 (10)	0.49113 (14)	0.21304 (16)	0.0282 (9)	0.780 (6)
C20A	0.12933 (15)	0.5332 (2)	0.0708 (2)	0.0250 (10)	0.780 (6)
H20A	0.1070	0.5806	0.0687	0.030*	0.780 (6)
H20B	0.1102	0.4830	0.0445	0.030*	0.780 (6)
C21A	0.15854 (14)	0.5148 (2)	0.1632 (3)	0.0282 (10)	0.780 (6)
H21A	0.1813	0.4682	0.1648	0.034*	0.780 (6)
H21B	0.1774	0.5654	0.1893	0.034*	0.780 (6)
O2B	0.0926 (3)	0.5060 (5)	0.1643 (5)	0.019 (2)*	0.220 (6)
C20B	0.1638 (5)	0.5335 (8)	0.1196 (10)	0.019 (2)*	0.220 (6)
H20C	0.1782	0.4778	0.1402	0.022*	0.220 (6)
H20D	0.1786	0.5782	0.1622	0.022*	0.220 (6)
C21B	0.1100 (4)	0.5333 (7)	0.0924 (7)	0.019 (2)*	0.220 (6)
H21C	0.0978	0.5908	0.0735	0.022*	0.220 (6)
H21D	0.0973	0.4946	0.0427	0.022*	0.220 (6)
C1S	0.0950 (4)	0.8005 (7)	0.2786 (7)	0.082 (2)*	0.25
H1S1	0.1110	0.8128	0.3399	0.122*	0.25
H1S2	0.1098	0.8340	0.2434	0.122*	0.25
H1S3	0.0984	0.7402	0.2679	0.122*	0.25
C2S	0.0488 (7)	0.8199 (13)	0.2578 (13)	0.082 (2)*	0.25
N2S	0.0000	0.8254 (7)	0.2500	0.082 (2)*	0.50

### Atomic displacement parameters (Å^2^)

**Table:**

	*U*^11^	*U*^22^	*U*^33^	*U*^12^	*U*^13^	*U*^23^
O1	0.0542 (14)	0.0113 (9)	0.0335 (11)	−0.0014 (8)	0.0307 (10)	−0.0023 (8)
O3	0.0843 (19)	0.0267 (13)	0.0502 (14)	−0.0171 (11)	0.0479 (14)	−0.0105 (10)
O4	0.0472 (13)	0.0229 (11)	0.0416 (12)	−0.0021 (9)	0.0320 (11)	−0.0020 (9)
O5	0.0417 (13)	0.0275 (11)	0.0486 (13)	−0.0024 (9)	0.0275 (11)	−0.0090 (9)
N1	0.0529 (17)	0.0207 (12)	0.0339 (15)	−0.0020 (11)	0.0264 (14)	0.0032 (11)
N2	0.096 (3)	0.0168 (15)	0.075 (2)	0.0010 (14)	0.065 (2)	0.0018 (13)
N3	0.0564 (18)	0.0185 (12)	0.0320 (14)	0.0034 (11)	0.0278 (13)	0.0006 (11)
N4	0.0481 (16)	0.0150 (12)	0.0299 (13)	−0.0032 (10)	0.0262 (12)	−0.0014 (10)
C1	0.0384 (17)	0.0120 (13)	0.0318 (16)	−0.0006 (12)	0.0190 (15)	0.0062 (12)
C2	0.0480 (19)	0.0128 (13)	0.0292 (15)	−0.0002 (12)	0.0261 (15)	−0.0002 (11)
C3	0.059 (2)	0.0188 (17)	0.0429 (19)	0.0017 (14)	0.0372 (17)	0.0035 (13)
C4	0.0392 (17)	0.0157 (14)	0.0232 (14)	−0.0017 (12)	0.0182 (13)	−0.0006 (11)
C5	0.0445 (18)	0.0148 (13)	0.0254 (15)	−0.0025 (12)	0.0226 (14)	0.0012 (11)
C6	0.0379 (17)	0.0176 (14)	0.0273 (15)	−0.0007 (12)	0.0185 (14)	−0.0025 (11)
C7	0.0391 (17)	0.0131 (13)	0.0246 (15)	−0.0012 (11)	0.0192 (13)	−0.0016 (11)
C8	0.051 (2)	0.0228 (15)	0.0374 (18)	0.0002 (13)	0.0306 (16)	−0.0021 (13)
C9	0.056 (2)	0.0209 (15)	0.0295 (17)	−0.0008 (14)	0.0217 (16)	−0.0040 (12)
C10	0.0417 (18)	0.0124 (13)	0.0253 (15)	−0.0008 (12)	0.0196 (14)	0.0043 (12)
C11	0.053 (2)	0.0178 (14)	0.0268 (15)	−0.0027 (13)	0.0268 (15)	−0.0001 (12)
C12	0.0448 (18)	0.0183 (14)	0.0241 (15)	−0.0001 (12)	0.0219 (14)	0.0010 (11)
C13	0.075 (2)	0.0164 (15)	0.0474 (19)	−0.0038 (15)	0.0483 (19)	−0.0002 (13)
C14	0.052 (2)	0.0167 (14)	0.0342 (17)	−0.0028 (13)	0.0292 (16)	−0.0011 (12)
C15	0.064 (2)	0.0126 (13)	0.0368 (17)	−0.0090 (13)	0.0352 (17)	−0.0036 (12)
C16	0.052 (2)	0.0177 (14)	0.0328 (16)	−0.0078 (13)	0.0293 (16)	−0.0025 (12)
C17	0.0458 (19)	0.0138 (13)	0.0318 (16)	−0.0041 (12)	0.0220 (15)	−0.0003 (12)
C18	0.0470 (19)	0.0186 (14)	0.0325 (16)	−0.0055 (13)	0.0268 (15)	−0.0003 (12)
C19	0.076 (3)	0.0174 (15)	0.050 (2)	−0.0004 (15)	0.045 (2)	−0.0058 (14)
C22	0.085 (3)	0.0296 (19)	0.044 (2)	−0.0158 (17)	0.047 (2)	−0.0121 (15)
C23	0.0440 (19)	0.0305 (16)	0.0248 (15)	−0.0070 (14)	0.0201 (15)	−0.0043 (13)
C24	0.0406 (18)	0.0285 (16)	0.0298 (16)	−0.0040 (13)	0.0236 (15)	−0.0066 (13)
C25	0.0327 (17)	0.0228 (15)	0.0269 (15)	−0.0044 (12)	0.0150 (14)	0.0004 (12)
C26	0.0322 (17)	0.0306 (16)	0.0300 (16)	−0.0060 (13)	0.0188 (14)	−0.0026 (13)
C27	0.0303 (17)	0.0283 (16)	0.0260 (15)	0.0002 (12)	0.0120 (14)	−0.0044 (12)
C28	0.0378 (18)	0.0256 (15)	0.0225 (15)	−0.0055 (13)	0.0131 (14)	−0.0007 (12)
C29	0.0403 (18)	0.0289 (16)	0.0316 (16)	0.0023 (13)	0.0223 (15)	−0.0007 (13)
C30	0.0345 (17)	0.0289 (16)	0.0278 (16)	0.0013 (13)	0.0163 (14)	−0.0012 (12)
C31	0.0413 (19)	0.0351 (17)	0.0341 (17)	0.0027 (14)	0.0216 (15)	−0.0026 (14)
C32	0.053 (2)	0.0279 (17)	0.046 (2)	0.0078 (15)	0.0244 (18)	−0.0046 (14)
C33	0.053 (2)	0.0254 (16)	0.045 (2)	−0.0016 (15)	0.0217 (18)	−0.0003 (14)
C34	0.0448 (19)	0.0296 (17)	0.0358 (17)	−0.0023 (14)	0.0229 (16)	0.0019 (13)
C35	0.0394 (18)	0.0291 (16)	0.0327 (17)	0.0048 (13)	0.0216 (15)	0.0001 (13)
C36	0.044 (2)	0.0261 (16)	0.049 (2)	0.0071 (14)	0.0245 (17)	−0.0001 (14)
C37	0.0404 (18)	0.0198 (14)	0.0454 (19)	0.0050 (13)	0.0314 (16)	−0.0027 (13)
C38	0.060 (2)	0.0276 (17)	0.051 (2)	0.0024 (16)	0.0357 (19)	0.0044 (15)
C39	0.063 (2)	0.0293 (18)	0.080 (3)	−0.0146 (17)	0.050 (2)	−0.0101 (18)
C40	0.048 (2)	0.043 (2)	0.054 (2)	−0.0058 (16)	0.0307 (19)	−0.0148 (18)
C41	0.0397 (19)	0.0334 (17)	0.0434 (19)	0.0032 (14)	0.0233 (16)	−0.0057 (15)
C42	0.0396 (18)	0.0209 (14)	0.0422 (18)	0.0020 (13)	0.0285 (16)	−0.0024 (13)
O2A	0.045 (2)	0.0226 (13)	0.0289 (16)	−0.0052 (12)	0.0292 (15)	−0.0001 (11)
C20A	0.039 (3)	0.0168 (17)	0.028 (2)	−0.0010 (16)	0.023 (2)	−0.0015 (15)
C21A	0.042 (2)	0.025 (2)	0.026 (2)	−0.0020 (17)	0.0231 (19)	−0.0012 (16)

### Geometric parameters (Å, °)

**Table:**

O1—C6	1.352 (3)	C24—H24	0.9500
O1—C5	1.478 (3)	C25—C26	1.385 (4)
O3—C22	1.212 (4)	C26—C27	1.382 (4)
O4—C25	1.372 (3)	C26—H26	0.9500
O4—C29	1.437 (3)	C27—C28	1.384 (4)
O5—C27	1.380 (3)	C28—H28	0.9500
O5—C36	1.426 (3)	C29—C30	1.500 (4)
N1—C1	1.157 (3)	C29—H29A	0.9900
N2—C3	1.152 (4)	C29—H29B	0.9900
N3—C10	1.149 (3)	C30—C35	1.390 (4)
N4—C17	1.347 (4)	C30—C31	1.393 (4)
N4—C16	1.348 (3)	C31—C32	1.386 (4)
N4—C19	1.475 (3)	C31—H31	0.9500
C1—C2	1.412 (4)	C32—C33	1.382 (5)
C2—C6	1.395 (4)	C32—H32	0.9500
C2—C3	1.417 (4)	C33—C34	1.374 (4)
C4—C11	1.366 (4)	C33—H33	0.9500
C4—C7	1.430 (4)	C34—C35	1.385 (4)
C4—C5	1.513 (4)	C34—H34	0.9500
C5—C9	1.516 (4)	C35—H35	0.9500
C5—C8	1.527 (4)	C36—C37	1.508 (5)
C6—C7	1.397 (4)	C36—H36A	0.9900
C7—C10	1.424 (4)	C36—H36B	0.9900
C8—H8A	0.9800	C37—C38	1.386 (4)
C8—H8B	0.9800	C37—C42	1.396 (4)
C8—H8C	0.9800	C38—C39	1.394 (5)
C9—H9A	0.9800	C38—H38	0.9500
C9—H9B	0.9800	C39—C40	1.378 (5)
C9—H9C	0.9800	C39—H39	0.9500
C11—C12	1.415 (4)	C40—C41	1.378 (5)
C11—H11	0.9500	C40—H40	0.9500
C12—C13	1.357 (4)	C41—C42	1.383 (4)
C12—H12	0.9500	C41—H41	0.9500
C13—C14	1.462 (4)	C42—H42	0.9500
C13—C20A	1.518 (5)	O2A—C21A	1.452 (5)
C13—C20B	1.643 (15)	C20A—C21A	1.507 (6)
C14—C18	1.410 (4)	C20A—H20A	0.9900
C14—C15	1.411 (4)	C20A—H20B	0.9900
C15—C16	1.361 (4)	C21A—H21A	0.9900
C15—H15	0.9500	C21A—H21B	0.9900
C16—H16	0.9500	O2B—C21B	1.486 (14)
C17—C18	1.362 (4)	C20B—C21B	1.498 (17)
C17—H17	0.9500	C20B—H20C	0.9900
C18—H18	0.9500	C20B—H20D	0.9900
C19—H19A	0.9800	C21B—H21C	0.9900
C19—H19B	0.9800	C21B—H21D	0.9900
C19—H19C	0.9800	C1S—C2S	1.33 (2)
C22—O2A	1.380 (4)	C1S—H1S1	0.9800
C22—O2B	1.470 (9)	C1S—H1S2	0.9800
C22—C23	1.488 (4)	C1S—H1S3	0.9800
C23—C24	1.390 (4)	C2S—N2S	1.40 (2)
C23—C28	1.391 (4)	N2S—C2S^i^	1.40 (2)
C24—C25	1.378 (4)		
			
C6—O1—C5	109.24 (19)	C27—C26—C25	120.1 (3)
C25—O4—C29	117.6 (2)	C27—C26—H26	119.9
C27—O5—C36	119.4 (2)	C25—C26—H26	119.9
C17—N4—C16	119.8 (2)	O5—C27—C26	114.3 (2)
C17—N4—C19	120.5 (2)	O5—C27—C28	124.8 (3)
C16—N4—C19	119.7 (2)	C26—C27—C28	120.9 (3)
N1—C1—C2	177.7 (3)	C27—C28—C23	117.8 (3)
C6—C2—C1	122.5 (2)	C27—C28—H28	121.1
C6—C2—C3	120.5 (2)	C23—C28—H28	121.1
C1—C2—C3	117.0 (2)	O4—C29—C30	109.3 (2)
N2—C3—C2	179.0 (3)	O4—C29—H29A	109.8
C11—C4—C7	130.3 (2)	C30—C29—H29A	109.8
C11—C4—C5	123.7 (2)	O4—C29—H29B	109.8
C7—C4—C5	106.0 (2)	C30—C29—H29B	109.8
O1—C5—C4	104.19 (19)	H29A—C29—H29B	108.3
O1—C5—C9	107.3 (2)	C35—C30—C31	118.7 (3)
C4—C5—C9	112.6 (2)	C35—C30—C29	122.9 (3)
O1—C5—C8	106.3 (2)	C31—C30—C29	118.4 (3)
C4—C5—C8	113.8 (2)	C32—C31—C30	120.4 (3)
C9—C5—C8	111.9 (2)	C32—C31—H31	119.8
O1—C6—C2	117.2 (2)	C30—C31—H31	119.8
O1—C6—C7	111.5 (2)	C33—C32—C31	120.3 (3)
C2—C6—C7	131.4 (2)	C33—C32—H32	119.9
C6—C7—C10	123.6 (2)	C31—C32—H32	119.9
C6—C7—C4	109.1 (2)	C34—C33—C32	119.7 (3)
C10—C7—C4	126.9 (2)	C34—C33—H33	120.1
C5—C8—H8A	109.5	C32—C33—H33	120.1
C5—C8—H8B	109.5	C33—C34—C35	120.4 (3)
H8A—C8—H8B	109.5	C33—C34—H34	119.8
C5—C8—H8C	109.5	C35—C34—H34	119.8
H8A—C8—H8C	109.5	C34—C35—C30	120.5 (3)
H8B—C8—H8C	109.5	C34—C35—H35	119.8
C5—C9—H9A	109.5	C30—C35—H35	119.8
C5—C9—H9B	109.5	O5—C36—C37	113.9 (2)
H9A—C9—H9B	109.5	O5—C36—H36A	108.8
C5—C9—H9C	109.5	C37—C36—H36A	108.8
H9A—C9—H9C	109.5	O5—C36—H36B	108.8
H9B—C9—H9C	109.5	C37—C36—H36B	108.8
N3—C10—C7	177.0 (3)	H36A—C36—H36B	107.7
C4—C11—C12	123.7 (2)	C38—C37—C42	118.1 (3)
C4—C11—H11	118.2	C38—C37—C36	120.7 (3)
C12—C11—H11	118.2	C42—C37—C36	120.9 (3)
C13—C12—C11	129.0 (3)	C37—C38—C39	120.6 (3)
C13—C12—H12	115.5	C37—C38—H38	119.7
C11—C12—H12	115.5	C39—C38—H38	119.7
C12—C13—C14	120.3 (3)	C40—C39—C38	120.5 (3)
C12—C13—C20A	120.6 (3)	C40—C39—H39	119.8
C14—C13—C20A	118.9 (3)	C38—C39—H39	119.8
C12—C13—C20B	112.8 (5)	C39—C40—C41	119.6 (3)
C14—C13—C20B	115.7 (5)	C39—C40—H40	120.2
C18—C14—C15	114.8 (2)	C41—C40—H40	120.2
C18—C14—C13	122.2 (2)	C40—C41—C42	120.1 (3)
C15—C14—C13	123.0 (2)	C40—C41—H41	119.9
C16—C15—C14	121.7 (3)	C42—C41—H41	119.9
C16—C15—H15	119.1	C41—C42—C37	121.1 (3)
C14—C15—H15	119.1	C41—C42—H42	119.4
N4—C16—C15	120.9 (3)	C37—C42—H42	119.4
N4—C16—H16	119.5	C22—O2A—C21A	116.9 (2)
C15—C16—H16	119.5	C21A—C20A—C13	108.7 (3)
N4—C17—C18	121.1 (2)	C21A—C20A—H20A	110.0
N4—C17—H17	119.4	C13—C20A—H20A	110.0
C18—C17—H17	119.4	C21A—C20A—H20B	110.0
C17—C18—C14	121.6 (3)	C13—C20A—H20B	110.0
C17—C18—H18	119.2	H20A—C20A—H20B	108.3
C14—C18—H18	119.2	O2A—C21A—C20A	110.6 (3)
N4—C19—H19A	109.5	O2A—C21A—H21A	109.5
N4—C19—H19B	109.5	C20A—C21A—H21A	109.5
H19A—C19—H19B	109.5	O2A—C21A—H21B	109.5
N4—C19—H19C	109.5	C20A—C21A—H21B	109.5
H19A—C19—H19C	109.5	H21A—C21A—H21B	108.1
H19B—C19—H19C	109.5	C22—O2B—C21B	118.6 (7)
O3—C22—O2A	123.0 (3)	C21B—C20B—C13	91.8 (9)
O3—C22—O2B	115.1 (4)	C21B—C20B—H20C	113.3
O2A—C22—O2B	45.3 (3)	C13—C20B—H20C	113.3
O3—C22—C23	125.3 (3)	C21B—C20B—H20D	113.3
O2A—C22—C23	111.4 (3)	C13—C20B—H20D	113.3
O2B—C22—C23	106.2 (4)	H20C—C20B—H20D	110.6
C24—C23—C28	122.1 (3)	O2B—C21B—C20B	111.3 (10)
C24—C23—C22	120.1 (3)	O2B—C21B—H21C	109.4
C28—C23—C22	117.8 (3)	C20B—C21B—H21C	109.4
C25—C24—C23	118.5 (3)	O2B—C21B—H21D	109.4
C25—C24—H24	120.7	C20B—C21B—H21D	109.4
C23—C24—H24	120.7	H21C—C21B—H21D	108.0
O4—C25—C24	124.6 (2)	C1S—C2S—N2S	166.3 (18)
O4—C25—C26	114.9 (2)	C2S^i^—N2S—C2S	172.9 (19)
C24—C25—C26	120.4 (3)		
			
C6—O1—C5—C4	−0.3 (3)	C28—C23—C24—C25	0.2 (5)
C6—O1—C5—C9	119.3 (2)	C22—C23—C24—C25	178.8 (3)
C6—O1—C5—C8	−120.8 (2)	C29—O4—C25—C24	−0.2 (4)
C11—C4—C5—O1	−178.4 (3)	C29—O4—C25—C26	179.8 (3)
C7—C4—C5—O1	1.6 (3)	C23—C24—C25—O4	179.8 (3)
C11—C4—C5—C9	65.7 (4)	C23—C24—C25—C26	−0.3 (4)
C7—C4—C5—C9	−114.4 (3)	O4—C25—C26—C27	−179.1 (3)
C11—C4—C5—C8	−63.0 (4)	C24—C25—C26—C27	0.9 (4)
C7—C4—C5—C8	116.9 (3)	C36—O5—C27—C26	175.3 (3)
C5—O1—C6—C2	178.2 (3)	C36—O5—C27—C28	−5.9 (4)
C5—O1—C6—C7	−1.2 (3)	C25—C26—C27—O5	177.3 (3)
C1—C2—C6—O1	178.3 (3)	C25—C26—C27—C28	−1.6 (5)
C3—C2—C6—O1	−0.8 (4)	O5—C27—C28—C23	−177.3 (3)
C1—C2—C6—C7	−2.5 (5)	C26—C27—C28—C23	1.5 (4)
C3—C2—C6—C7	178.4 (3)	C24—C23—C28—C27	−0.9 (5)
O1—C6—C7—C10	174.9 (3)	C22—C23—C28—C27	−179.4 (3)
C2—C6—C7—C10	−4.3 (5)	C25—O4—C29—C30	−178.0 (2)
O1—C6—C7—C4	2.3 (4)	O4—C29—C30—C35	14.4 (4)
C2—C6—C7—C4	−177.0 (3)	O4—C29—C30—C31	−167.4 (3)
C11—C4—C7—C6	177.6 (3)	C35—C30—C31—C32	−0.8 (5)
C5—C4—C7—C6	−2.3 (3)	C29—C30—C31—C32	−179.1 (3)
C11—C4—C7—C10	5.3 (5)	C30—C31—C32—C33	0.5 (5)
C5—C4—C7—C10	−174.7 (3)	C31—C32—C33—C34	0.2 (5)
C7—C4—C11—C12	6.6 (5)	C32—C33—C34—C35	−0.5 (5)
C5—C4—C11—C12	−173.4 (3)	C33—C34—C35—C30	0.1 (5)
C4—C11—C12—C13	−170.8 (3)	C31—C30—C35—C34	0.5 (5)
C11—C12—C13—C14	−177.2 (3)	C29—C30—C35—C34	178.7 (3)
C11—C12—C13—C20A	8.4 (6)	C27—O5—C36—C37	−78.9 (3)
C11—C12—C13—C20B	−35.0 (7)	O5—C36—C37—C38	152.5 (3)
C12—C13—C14—C18	175.4 (3)	O5—C36—C37—C42	−34.0 (4)
C20A—C13—C14—C18	−10.1 (5)	C42—C37—C38—C39	−1.9 (4)
C20B—C13—C14—C18	34.2 (7)	C36—C37—C38—C39	171.8 (3)
C12—C13—C14—C15	−5.0 (5)	C37—C38—C39—C40	1.2 (5)
C20A—C13—C14—C15	169.6 (3)	C38—C39—C40—C41	1.1 (5)
C20B—C13—C14—C15	−146.1 (6)	C39—C40—C41—C42	−2.6 (5)
C18—C14—C15—C16	−1.5 (5)	C40—C41—C42—C37	1.9 (4)
C13—C14—C15—C16	178.8 (3)	C38—C37—C42—C41	0.4 (4)
C17—N4—C16—C15	−0.6 (5)	C36—C37—C42—C41	−173.3 (3)
C19—N4—C16—C15	179.9 (3)	O3—C22—O2A—C21A	−3.8 (5)
C14—C15—C16—N4	1.8 (5)	C23—C22—O2A—C21A	−177.8 (3)
C16—N4—C17—C18	−0.8 (4)	C12—C13—C20A—C21A	−93.6 (4)
C19—N4—C17—C18	178.7 (3)	C14—C13—C20A—C21A	91.9 (4)
N4—C17—C18—C14	1.0 (5)	C22—O2A—C21A—C20A	−82.2 (4)
C15—C14—C18—C17	0.2 (5)	C13—C20A—C21A—O2A	−179.1 (2)
C13—C14—C18—C17	179.9 (3)	O3—C22—O2B—C21B	26.8 (9)
O3—C22—C23—C24	−176.7 (4)	C23—C22—O2B—C21B	170.0 (7)
O2A—C22—C23—C24	−2.8 (5)	C12—C13—C20B—C21B	104.1 (7)
O2B—C22—C23—C24	45.0 (5)	C14—C13—C20B—C21B	−111.9 (7)
O3—C22—C23—C28	1.9 (6)	C22—O2B—C21B—C20B	60.6 (11)
O2A—C22—C23—C28	175.8 (3)	C13—C20B—C21B—O2B	174.5 (7)
O2B—C22—C23—C28	−136.4 (4)		

Symmetry codes: (i) −*x*, *y*, −*z*+1/2.

### Hydrogen-bond geometry (Å, °)

**Table:**

*D*—H···*A*	*D*—H	H···*A*	*D*···*A*	*D*—H···*A*
C9—H9A···N1^ii^	0.98	2.59	3.529 (4)	161
C9—H9B···N3^iii^	0.98	2.59	3.497 (5)	153
C16—H16···N1^iv^	0.95	2.51	3.406 (4)	156
C17—H17···N2^v^	0.95	2.51	3.380 (4)	152
C19—H19C···N2^v^	0.98	2.50	3.340 (4)	143
C26—H26···O5^vi^	0.95	2.51	3.398 (4)	155
C8—H8B···Cg1^vii^	0.98	2.54	3.515 (3)	171

Symmetry codes: (ii) *x*, −*y*+2, *z*+1/2; (iii) −*x*+1/2, −*y*+3/2, −*z*; (iv) −*x*+1/2, *y*−1/2, −*z*−1/2; (v) *x*, *y*−1, *z*; (vi) −*x*, −*y*+1, −*z*+1; (vii) *x*, −*y*+1, *z*−1/2.

### Table 2 Interplanar angles of the planar entities (°)

**Table:**

Plane	C1–C12,N1–N3,O1	C12–C19,N4	C22–C30,O3–O4	C29–C35	C37–C42	SIGP^a^,Å
C1–C12,N1–N3,O1		14.59 (10)	18.77 (7)	31.92 (12)	64.58 (13)	0.025 (3)
C12–C19,N4	14.59 (10)		4.90 (9)	18.33 (13)	75.25 (15)	0.033 (3)
C22–C30,O3–O4	18.77 (7)	4.90 (9)		13.48 (11)	80.11 (12)	0.026 (3)
C29–C35^b^	31.92 (12)	18.33 (13)	13.48 (11)		86.94 (16)	0.004 (3)

Notes: (a) Sqrt(Sum(j=1:N)(D(j)**2/(N-3)) (Spek, 2009); (b) SIGP for plane C37–C42 is 0.014 (3) Å.
